# Advances for the Hepatitis A Virus Antigen Production Using a Virus Strain With Codon Frequency Optimization Adjustments in Specific Locations

**DOI:** 10.3389/fmicb.2021.642267

**Published:** 2021-02-18

**Authors:** Gemma Chavarria-Miró, Montserrat de Castellarnau, Cristina Fuentes, Lucía D’Andrea, Francisco-Javier Pérez-Rodríguez, Nerea Beguiristain, Albert Bosch, Susana Guix, Rosa M. Pintó

**Affiliations:** Enteric Virus Laboratory, Department of Genetics, Microbiology and Statistics, School of Biology, Institute of Nutrition and Food Safety, Campus Torribera, University of Barcelona, Barcelona, Spain

**Keywords:** hepatitis A, HAV, codon composition, IRES, vaccine, FRhK-4, MRC-5, Vero

## Abstract

The available cell-adapted hepatitis A virus (HAV) strains show a very slow replication phenotype hampering the affordable production of antigen. A fast-growing strain characterized by the occurrence of mutations in the internal ribosome entry site (IRES), combined with changes in the codon composition has been selected in our laboratory. A characterization of the IRES activity of this fast-growing strain (HM175-HP; HP) vs. its parental strain (HM175; L0) was assessed in two cell substrates used in vaccine production (MRC-5 and Vero cells) compared with the FRhK-4 cell line in which its selection was performed. The HP-derived IRES was significantly more active than the L0-derived IRES in all cells tested and both IRES were more active in the FRhK-4 cells. The translation efficiency of the HP-derived IRES was also much higher than the L0-derived IRES, particularly, in genes with a HP codon usage background. These results correlated with a higher virus production in a shorter time for the HP strain compared to the L0 strain in any of the three cell lines tested, and of both strains in the FRhK-4 cells compared to Vero and MRC-5 cells. The addition of wortmannin resulted in the increase of infectious viruses and antigen in the supernatant of FRhK-4 infected cells, independently of the strain. Finally, the replication of both strains in a clone of FRhK-4 cells adapted to grow with synthetic sera was optimal and again the HP strain showed higher yields.

## Introduction

Hepatitis A outbreaks are on the rise. Recently, a huge outbreak has hit the men-having-sex-with-men (MSM) group across Europe with over 4,000 cases (Beebeejaun et al., 2017; Freidl et al., 2017; Werber et al., 2017; Ecdc., 2018). Likewise, in the US, outbreaks among people who use drugs, people experiencing homelessness and the MSM group, have acquired vast dimensions with over 30,000 cases and 300 casualties^1^.

The control of these outbreaks has been hampered by low vaccination coverage and by vaccine shortages, which have sometimes been overcome by restricting the number of vaccine doses and their content. However, while these measures attempt to avoid as much as possible the spread of the outbreaks and to protect the patients’ health, they may prompt the selection of vaccine-escape virus variants (Pintó and Bosch, 2019; Sabrià et al., 2019).

Vaccine shortages may be due to higher-than-expected demand and/or interruptions in supply caused by production problems (Hinman et al., 2006). Production of large amounts of antigens, required for inactivated vaccines, is still a critical step, particularly for viruses with poor growth in cell culture such as the hepatitis A virus (HAV) (Lemon et al., 1992). Several strains of HAV have been adapted to replicate *in vitro*, including the HM-175, CR-326, GBM, TZ84, Lv-8, YN5, and RG-SB strains (Lemon et al., 2017), but production of industrial batches require large scale-ups resulting in very high costs.

The slow growth of HAV in cell culture is the consequence of the combination of at least three factors: its inefficient internal ribosome entry site (IRES) in directing translation (Brown et al., 1994; Whetter et al., 1994), its inability to induce the cellular protein synthesis shutoff (Ali et al., 2001), and its deviated codon usage with respect to the cell codon usage (Aragonès et al., 2010; Pintó et al., 2018). Altogether may result in competition for the translational machinery and tRNAs, which additionally may not be well adapted to the virus requirements, contributing to the modestly productive growth of HAV in cell culture.

A fast-growing strain of HAV, termed HM-175-HP, derived from the HM-175 strain has been recently selected (Pérez-Rodríguez et al., 2016). The HM-175 strain was adapted to grow in conditions of artificially induced transcription shutoff by treating the cells with actinomycin D (AMD), and two populations long adapted to moderate and high levels of shutoff were selected (D’Andrea et al., 2019). Competition experiments between these two populations, enabled the rescue of the HM-175-HP clone (Pérez-Rodríguez et al., 2016). This strain acquired three mutations in the IRES and changed the codon frequency particularly in the VP1 capsid coding region. The IRES became more active, and the new codons were optimized with respect the codon usage. The complete selection process was performed on the FRhK-4 (Fetal Rhesus kidney) cell line.

Most HAV inactivated vaccines are produced in human embryonic diploid fibroblasts (MRC-5, 2BS, and KMB17 cells) or in continuous cell lines derived from the African green monkey (Vero cells). The World Health Organization (WHO) guidelines on vaccine production standardization^2^ recommend testing for the presence of adventitious agents in the cells as well as in sera or any other reagent susceptible to be contaminated, such as the trypsin. Additionally, testing for oncogenicity and tumorigenicity is also a recommendation. The vaccine based in the HM-175 strain is produced in MRC-5 cells, whose seed stocks production was overseen by the WHO Expert Committee on Biological Standardization and are in the WHO Reference Cell Bank. Nevertheless, these stocks are diminishing and due to both technical and ethical issues, it is extremely difficult to derive novel human diploid fibroblasts from fetal lung tissues (Chen et al., 2017). The continuous Vero cell line could also be used, but it has been suggested that tumorigenesis may be induced at high passages (Andreani et al., 2017).

The different cell substrates are associated with variations in the efficiency of the final product purification, and steps for removal of residual cellular constituents are especially important. In fact, the HAV antigen used for vaccine production is usually obtained from cell lysates since it is not efficiently released into the culture supernatant (Bishop et al., 1994). However, since the discovery of the HAV egress pathway (Feng et al., 2013), new strategies to improve the virus release into the supernatant may be designed.

In this study, we present data on the comparative translation rate, growth and virus release to the supernatant of the HM-175 and HM-175-HP strains in different cell substrates, which may have important implications for the improvement of HAV antigen production.

## Materials and Methods

### Cells and Viruses

Three different cell lines were used throughout this study: FRhK-4 cells (Fetal Rhesus kidney epithelial), MRC-5 cells (Medical Research Council cell strain 5; human diploid lung fibroblasts), and Vero cells (African green monkey kidney epithelial). These cells were grown in minimum essential medium (MEM) supplemented with fetal bovine serum (FBS) at concentrations of 15, 10, and 5%, for the FRhK-4, MRC-5, and Vero lines, respectively. The post-infection (p.i.) media contained 2% FBS.

Two previously characterized HAV strains were used throughout this study: HM-175-L0 (L0 thereafter) adapted to grow in the normal conditions of no cellular shutoff, and HM-175-HP (HP thereafter) adapted to grow in conditions of moderate transcription shutoff (Costafreda et al., 2014; Pérez-Rodríguez et al., 2016). Both populations were derived from the HM-175 strain (Gust et al., 1985).

Viral stocks were regularly produced in FRhK-4 cells using a multiplicity of infection (MOI) of 0.1. For HP, 0.05 μg/ml of AMD (Sigma), was added in the p.i. media to induce a 60–70% inhibition of the cell DNA transcription (Aragonès et al., 2010).

Virus infectious titers were obtained by infecting microtiter plates of FRhK-4 cells with serial 10-fold dilutions of virus supernatants in the absence of AMD (Costafreda et al., 2014). The plates were incubated at 37°C in a CO_2_ incubator and the development of HAV-induced cytopathic effect (CPE) was observed for 10 days. Titers were expressed as the Tissue Culture Infectious Dose 50 (TCID_50_) per ml of supernatant.

### *In vitro* Assays for the Determination of the IRES Activity and the Translation Efficiency

The bicistronic vector G1RC bears the *Renilla reniformis* luciferase gene (*RLuc*), whose translation is cap-dependent, and the *Photinus pyralis* (firefly) luciferase gene (*FLuc*), whose translation is dependent on the HAV IRES (Mackiewicz et al., 2010). The G1RC vector was modified by directed mutagenesis to include two restriction sites (*Msc*I and *Kpn*I) to allow the cloning of genes just after the IRES and before the *FLuc* gene (Supplementary Figure S1), and the new plasmid was called G1RCMsKp (Pérez-Rodríguez et al., 2016). The G1RCMsKp vector was further modified through the introduction of the mutations required to match the L0 IRES and subsequently with the mutations identifying the HP IRES (D’Andrea et al., 2019). These latter vectors were used to determine the IRES activity. Additionally, a fragment of the VP1 coding region was cloned in each of these vectors, just upstream of the *FLuc* gene and under the IRES translation control, and was used to determine the efficiency of translation. This VP1 fragment corresponded to the 50% of the total VP1 length, spanning nucleotides 2,394–2,852, differing in its codon composition between the L0 and the HP strains (D’Andrea et al., 2019), and with the highest Relative Codon Deoptimization Index deviation between both strains (Pérez-Rodríguez et al., 2016). Due to the low activity of the HAV IRES, we focused on this fragment to technically improve the detection of translation efficiency differences.

Monolayers of cells grown in 96 well microtiters were transfected with the different vectors. DNA was resuspended in Opti-MEM I (Thermo Fisher Scientific) at a concentration of 0.01 μg/μl and X-tremeGENE HP DNA Transfection Reagent (Roche) was added at a 4% (v:v) concentration. After incubating for 15 min at room temperature, 25 μl of this suspension was added to each well. Cells were incubated for a further 30 min at room temperature and finally 60 μl of post-transfection medium (Opti-MEM I) were added. After 24 hours the bioluminescence activity was measured with the Dual-Glo Luciferase Assay System (Promega) and detected with a luminometer (Lumat LB 9507, Berthold Technologies). Light emission was measured 10 min after addition of each substrate and integrated over a 10 s interval.

Three different experiments, each including two replicas, were performed for the determination of both the IRES activity and the translation efficiency (in the absence and presence of 0.05 μg/ml of AMD), respectively, and the results were figured as the mean and standard error of the FLuc/RLuc ratio of the six determinations. As negative controls, cells transfected with the digested vector alone were included. The ratio FLuc/RLuc was used to balance differences on transfection efficiency, transcription rate and mRNA export efficacy of the different vectors.

### Growth Curves

Virus growth curves were performed in FRhK-4 and MRC-5 cells incubated at 37°C using a multiplicity of infection (MOI) of 0.1. The HAV growth kinetics in Vero cells was performed at the permissive temperature of 33°C, using a MOI of 5. Additionally, the replication curve in the FRhK-4-BK cell-clone adapted to grow in synthetic sera, was also performed using MOIs of 0.01, 0.1, and 5.

The growth curves of the HP strain were made in the presence of 0.05 μg/ml AMD in all cell substrates except for the MRC-5 cell line.

### Induction of Virus Release Into the Cell Supernatant of FRhK-4 Cells

FRhK-4 cells were infected using a MOI of 1. At 24 h p.i., monolayers were washed twice with PBS, and fresh medium containing bafilomycin or wortmaninn (Sigma-Aldrich) at concentrations of 60 and 50 nM, respectively, was added; wortmannin was refreshed every 6 h. Drug concentrations were the highest granting an 80% cell viability. In parallel, monolayers of infected cells were identically processed but the refreshing medium did not contain any drug. Twelve hours after the addition of the drugs (36 h p.i.), supernatants from infected-untreated and infected-treated cells were collected, and viral titers were determined by TCID_50_ as above described. Three experiments with two replicas each were performed.

Additionally, the kinetics of virus release in the presence of wortmaninn was ascertained at longer times (12, 24, and 48 h of treatment), following the same procedure and refreshing the drug every 6 h.

### Sucrose-Iodixanol Gradients

Supernatants from infected cell cultures were centrifuged at 1,500 g for 10 min at 4°C to pellet any cellular debris, and clarified by centrifuging twice at 10,000*g* for 30 min at 4°C. Viruses were concentrated by ultracentrifugation at 100,000*g* for 1 h at 4°C. The pellets were resuspended in 1 ml of PBS and loaded onto pre-formed 6–50% iodixanol-(OptiPrep, Axis-Shield) sucrose step gradients, which were centrifuged at 205,000*g* for 2 h and 45 min at 4°C (SW41 Ti rotor in a Beckman Coulter Optima L-90K centrifuge). Approximately 20 fractions of 0.5 ml each were collected from the gradients and the density of each fraction was determined using a refractometer.

RNA from gradient fractions was extracted using the Nucleospin RNA Virus Extraction Kit (Macherey-Nagel) and HAV genome copy numbers were determined by a previously described Real-Time RT-PCR of the 5’ non-coding region (Costafreda et al., 2006), using the RNA UltraSense One-Step Quantitative RT-PCR System (Invitrogen).

### HAV Antigen Detection

L0 and HP antigens in the supernatants of infected cells in the presence or absence of wortmannin were analyzed by direct sandwich ELISA. Supernatants were treated with 1% NP-40 for 30 min at 37°C and three 30 s sonication cycles at 60 W. Mock-infected FRhK-4 cell supernatants identically processed were used as negative controls.

A human convalescent-phase serum was used for both capture (unlabeled antibodies) and detection of the HAV capsids (peroxidase-labeled antibodies). A cut-off level was established corresponding to the mean ±3*SD* of the unspecific recognition of mock-infected FRhK-4 cell supernatants.

Three different virus stocks of the L0 and HP strains produced in the presence or absence of wortmannin and three replicas of each stock were analyzed. The percentage of positive replicas for each stock and the number of ELISA units in each positive replica estimated.

### Statistical Analysis

Statistical differences between the IRES activity of the L0 and HP types in the different cell lines, the translation efficiency of the L0 and HP IRES types in the different codon usage backgrounds and in the presence and absence of AMD, the virus release of the L0 and HP virus populations in the presence or absence of drugs, and the convalescent-phase serum recognition of the L0 and HP capsids were assessed using the Student’s *t*-test (unpaired two-tailed) after verifying the normality of data with the Kolmogorov-Smirnov test. When normality failed the Mann-Whitney Rank Sum Test (SigmaPlot version 10) was applied. The difference of the IRES activity of a particular type in the different cells lines was tested with the One way ANOVA (SigmaPlot version 10).

## Results

### Activity of the Internal Ribosome Entry Site (IRES) Derived From the HM-175 (L0) and HM-175-HP (HP) Strains in Different Cell Substrates as an Indication of Virus Growth

It has been described that the replication rate of HAV, in some cell lines, is controlled to a significant degree by the efficiency of translation, particularly by the Internal Ribosome Entry Site (IRES) activity (Funkhouser et al., 1999).

**FIGURE 1:**
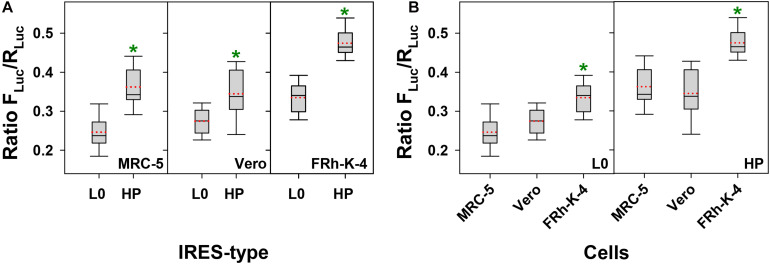
Comparative activity of L0 and HP IRES-types in MRC-5, Vero, and FRhK-4 cell lines. In the box plot diagrams, the box represents the 25th, 50th, and 75th percentiles, the whiskers the 5th and 95th percentiles, and the red dashed line the mean. **(A)** Activity of L0 and HP IRES-types in each cell line. *Significant statistical differences (*p* < 0.001, *p* = 0.002 and *p* < 0.001, in MRC-5, Vero, and FRhK-4 cells, respectively), assessed by the Student’s *t*-test. **(B)** Comparative activity of L0 IRES-type and HP IRES-type in each of the three cell lines. *Significant statistical differences (*p* < 0.001) assessed by the One-way ANOVA test.

In an attempt to identify the best cell substrate for the production of high antigen yields, we tested the activity of the L0- and HP-derived IRES, in three different cell lines: MRC-5, Vero and FRhK-4. The HP-derived IRES was significantly more efficient than the L0-derived in directing translation in all cells tested (*p* < 0.001, *p* = 0.002 and *p* < 0.001, in MRC-5, Vero and FRhK-4 cells, respectively) (Figure 1A). The average increase of the IRES activity between the HP and L0 was of around 1.5, 1.1, and 1.4 in MRC-5, Vero and FRhK-4 cells, respectively. Additionally, independently of the IRES-type, the translation efficiency was significantly higher in FRhK-4 cells (*p* < 0.001) (Figure 1B).

### Efficiency of the L0- and HP-Derived IRES in Directing the Translation of Capsid Regions With Different Codon Usage Backgrounds in the FRhK-4 Cells

The fast-growing phenotype of the HP strain is likely due to the inter-connected action of: (i) the mutations in the IRES, (ii) the right combination of codons in the capsid region, and (iii) the higher availability of resources in conditions of AMD induced transcription shutoff. To confirm the role of each of these factors we comparatively determined the translation efficiency after inter-changing the L0- and HP-derived IRES in codon usage backgrounds from the L0 and HP strains, in conditions of absence and presence of AMD. We took advantage of four bicistronic vectors (L0-IRES with a VP1 genome fragment from the L0 strain, HP-IRES with a VP1 genome fragment from the HP strain, L0-IRES with a VP1 genome fragment from the HP strain and HP-IRES with a VP1 genome fragment from the L0 strain) which were transfected into FRhK-4 cells.

**FIGURE 2:**
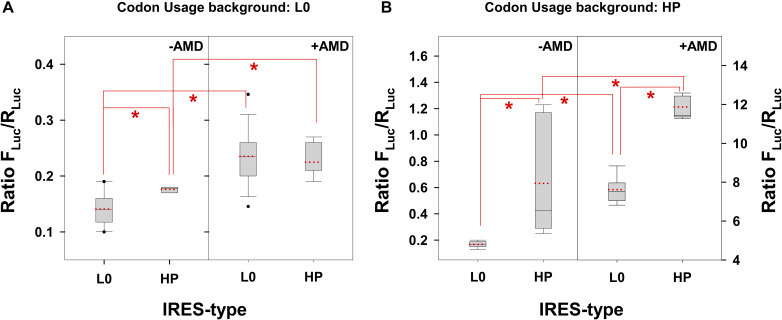
Translation efficiency of a fragment of the VP1 gene, whose codon composition is different in the L0 and HP strains, under the control of L0 and HP IRES-types and in the absence or presence of actinomycin D (AMD) in FRhK-4 cells. In the box plot diagrams, the box represents the 25th, 50th, and 75th percentiles, the whiskers the 5th and 95th percentiles, and the red dashed line the mean. **(A)** Translation efficiency of the VP1 fragment from the L0 strain. *Significant statistical differences (*p* < 0.001) assessed by the Student’s *t*-test. **(B)** Translation efficiency of the VP1 fragment from the HP strain. *Significant statistical differences (*p* < 0.001) assessed by the Student’s *t*-test.

Comparing the IRES-type effects, it could be concluded that the HP-derived IRES significantly (*p* < 0.001) increased (1.3 X) the translation of a VP1 genome fragment with a L0 codon usage background only in the absence of AMD (Figure 2A). Additionally, this increase was even more relevant and significant when translating the same VP1 fragment but with an HP codon usage background, in both the absence (3.4 X) and presence (1.6 X) of AMD (Figure 2B) with a *p* < 0.001. Finally, comparing exclusively the effect of the addition of AMD, there was a significant (*p* < 0.001) increase of the translation efficiency in all cases; however the magnitude of such increase was much more relevant in the HP codon usage background.

The actual virus strains have a L0-type IRES with a L0 codon usage background (L0 strain) and a HP-type IRES with a HP codon usage background (HP strain). In this scenario, the single factor to play with to increase the antigen yield is the addition of AMD. While the HP translation efficiency showed an increase of 18 X in the presence of AMD (*p* < 0.0001), in L0 this increase was of only 1.64 X (*p* < 0.001). Overall, the HP strain in the presence of AMD shows an efficiency of translation 100X higher (*p* < 0.001) compared to the L0 strain in the absence of AMD (Figure 2).

These results confirm that the HP strain grown in FRhK-4 cells in the presence of AMD is of great potential for antigen production.

### Comparative Virus Production of the L0 and HP Strains in MRC-5, Vero and FRhK-4 Cells

Virus productivity of L0 and HP strains in MRC-5 and FRhK-4 cells after infecting them at 37°C and a MOI of 0.1 was compared. L0 strain was grown in the absence of AMD, while the HP strain was grown in the absence of AMD in MRC-5 cells and in the presence of AMD in FRhK-4 cells.

**FIGURE 3:**
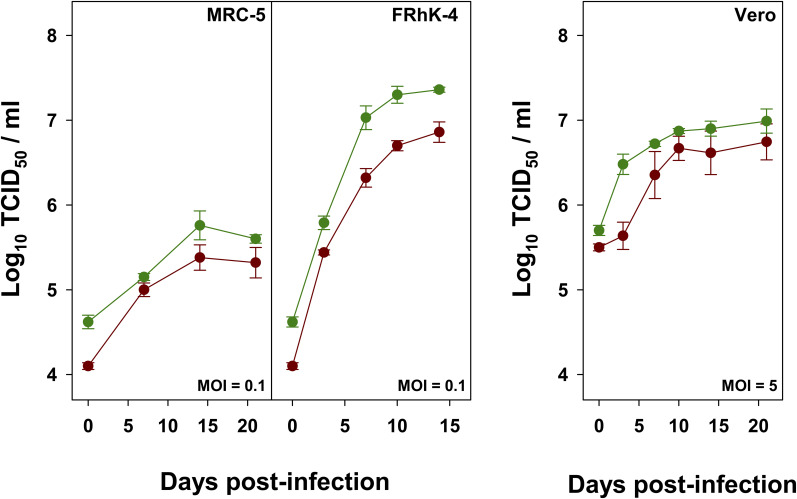
Growth curves of L0 (red) and HP (green) strains in MRC-5, FRhK-4 and Vero cells.

As expected from the IRES activity results, the highest yields in the shortest time were obtained using the FRhK-4 cell line, while the lowest yields were observed in the MRC-5 cells (Figure 3). Compared to L0, the HP strain showed maximum titer increases of 0.28 and 0.53 Log_10_ TCID_50_/ml in MRC-5, and FRhK-4 cells, respectively.

Replication in Vero cells at the permissive temperature of 33°C was also analyzed. L0 and HP strains were grown in the absence or presence of AMD, respectively, using a MOI of 5. Yields of the HP strain were also higher (Figure 3), with a maximum average increase of 0.25 Log_10_ TCID_50_/ml.

### Virus Release Into the Supernatants From L0 and HP-Infected FRhK-4 Cells

HAV antigens for vaccine and serology test kits production are commonly obtained by lysis of infected cells with detergents, followed by a concentration/purification step. However, the recent discoveries describing that HAV egress from cells as quasi-enveloped virions (eHAV) (Feng et al., 2013), i.e., capsids inside exosomes (McKnight et al., 2017), opens the possibility to improve antigen production by using culture supernatants instead of cell extracts.

In an attempt to increase the release of eHAV virions into the cell culture supernatant, we added bafilomycin or wortmannin at 24 h, p.i., to interfere with the traffic pathways, and analyzed the eHAV infectious titers in cell supernatants after 12 h of treatment. While bafilomycin inhibited the release of eHAV particles (fold increase of 0.31 ± 0.24 and 0.82 ± 0.32 for the L0 and HP strains, respectively), wortmannin improved their release (fold increase of 2.14 ± 0.83 and 5.24 ± 0.56 for the L0 and HP strains, respectively).

**FIGURE 4:**
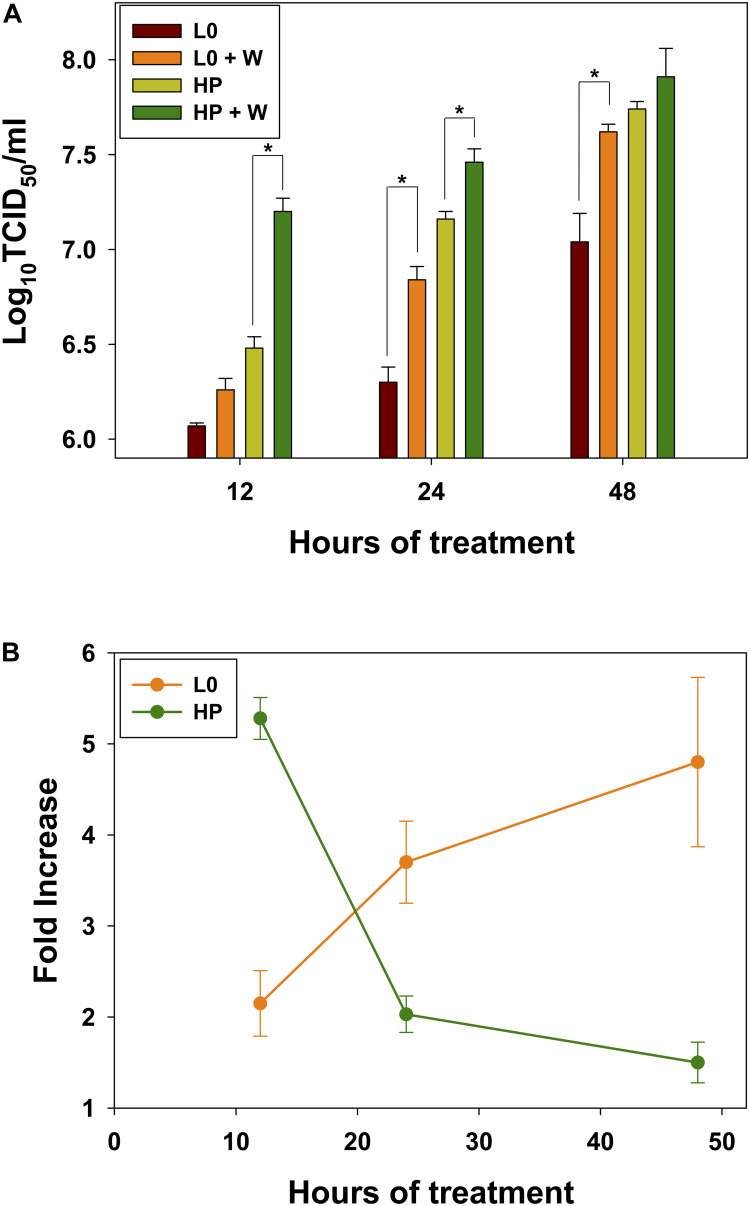
Effect of wortmannin on virus release into supernatants of FRhK-4 cells. **(A)** Virus titers of L0 and HP strains in the absence and presence of wortmannin (W). **(B)** Fold increase in virus titers in the supernatants with wortmannin treatment.

Additionally, we analyzed the effect of wortmannin after longer treatments (Figure 4). Wortmannin induced a significant increase of the eHAV egress, for the L0 population only after 24 h (*p* = 0.006) and 48 h (*p* = 0.016) treatments (Figure 4A). In contrast, for the HP population the egress significantly increased at shorter treatment times, of 12 h (*p* < 0.001) and 24 h (*p* = 0.026) (Figure 4A). The average fold-increases were of 2.15, 3.70, and 4.80 for L0 population and of 5.28, 2.03, and 1.5 for the HP population at 12, 24, and 48 h of treatment, respectively (Figure 4B).

**FIGURE 5:**
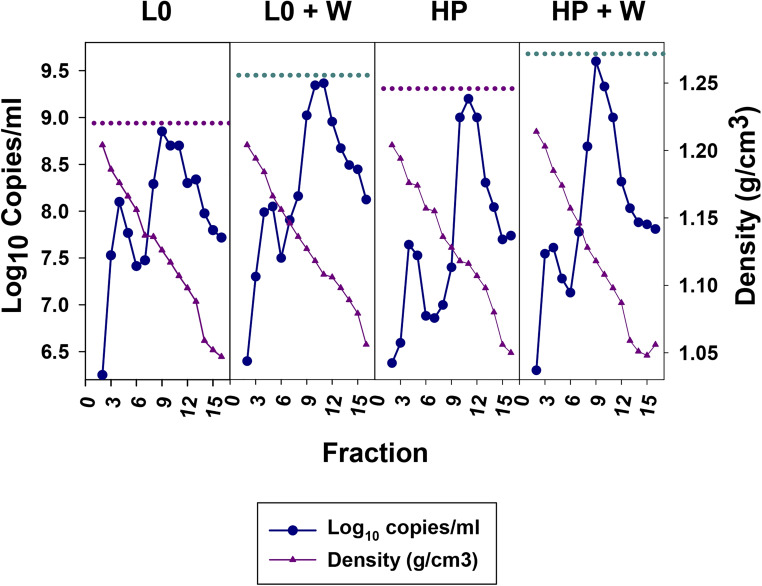
Sucrose-Iodixanol gradients of viruses present in the supernatant of FRhK-4 cells infected with the L0 and HP strains in the presence and absence of wortmannin (W). Naked and quasi-enveloped particles have densities of 1.20–1.25 and 1.06–1.12 g/cm^3^, respectively. The highest number of genome copies of quasi-enveloped particles in the absence or presence of wortmannin are depicted by dashed pink and cyan lines, respectively.

The effect of the wortmannin was further ascertained through the analysis of iodixanol-sucrose gradients of culture supernatants after 48 and 24 h of treatment for the L0 and HP populations, respectively. In both cases there was a clear increase of the genome copy numbers in the fractions with densities of 1.10–1.13 g/cm^3^, which correspond to the eHAV virions, after the wortmannin treatment. In the specific gradient shown in Figure 5, increases of around 5.0X and 2.5X were estimated for the L0 and HP populations at 48 and 24 h, respectively. These results indicate that the increments of infectivity observed in the supernatant of wortmannin-treated cells, are indeed due to an improved egress of the eHAV particles.

**FIGURE 6:**
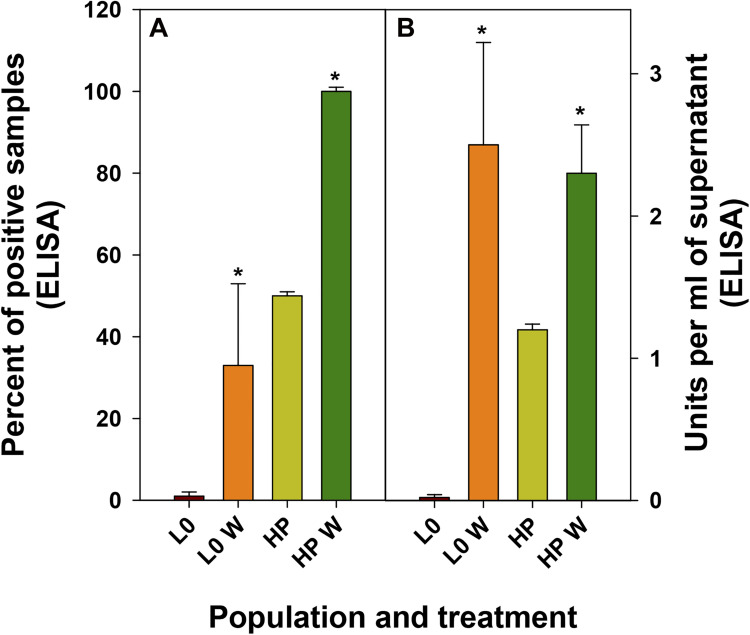
Antigen detection in supernatants of FRhK-4 infected cells in the absence or presence of wortmannin (W). **(A)** Mean percentage of replicas positive by an ELISA detecting the HAV antigen. **(B)** Mean of ELISA units present in the positive samples.

Finally, we evaluated how the improvement of eHAV release into the supernatants turned out in antigen yields. Again, wortmannin treatment significantly increased (*p* < 0.001) both, the number of positive supernatant replicas and the number of ELISA units in the positive replicas, independently of the population (Figure 6).

### Growth Curves of the L0 and HP Strains in FRhK-4 Cells Adapted to Synthetic Sera

The most expensive reagent to produce viruses in cell culture is fetal calf serum (FBS), particularly when using substrates such as the FRhK-4 cell line which require a supplement of 15% FBS.

**FIGURE 7:**
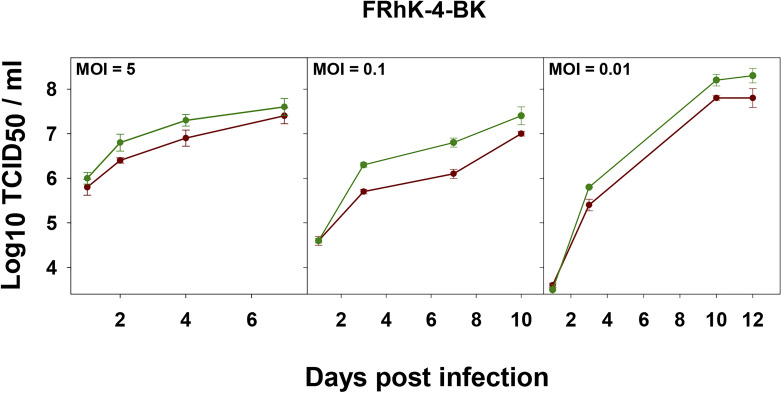
Growth curves of L0 (red) and HP (green) strains in FRhK-4-BK cells at different MOIs.

From data obtained in this study, we can conclude that the FRhK-4 cell substrate is the most permissive for the L0 and HP strains replication. To make the production more affordable, we tested the replication on a clone of the FRhK-4 cell line adapted to grow in the presence of synthetic sera (FRhK-4-BK cells). Growth kinetics for both populations was similar in FRhK-4 cells grown with either synthetic sera (Figure 7) or FBS (Figure 3).

As expected, the higher the MOI the shorter the time to achieve the maximum yields. Instead, a MOI as low as 0.01 combined with a replication time as long as 12 days yielded the highest titers (Figure 7). The HP population did show higher yields than the L0 population, particularly relevant when using a MOI of 0.01 (Figure 7).

## Discussion

Genome recoding approaches have lately been used to attenuate virus populations (Le Nouen et al., 2017, 2019), including codon deoptimization (Cheng et al., 2015), codon-pair deoptimization (Li et al., 2018; Mueller et al., 2020) and one-to-stop codons (Moratorio et al., 2017; Carrau et al., 2019). The basis of attenuation is the lower replication associated to a less efficient translation, the increased cellular antiviral responses associated to increases of CpG and UpA contents, and the lower robustness of the populations.

While these strategies are applicable to many different viruses, HAV is an exception to the rule. HAV has a naturally deoptimized codon usage (Sánchez et al., 2003), which contributes to a low replication phenotype (D’Andrea et al., 2019). The underlying basis of its deviated codon usage is the need for a finely tuned translation of the capsid coding region, which requires the precise location of clusters of rare codons (Aragonès et al., 2010). Rare codons in the HAV genome belong to two types: truly rare (in both the virus and the cell genomes) and rare by competition (rare in the virus genome but abundant in the cell genome). Since HAV is not able to shutdown the cellular protein synthesis it would compete for the tRNAs and the abundant tRNAs, pairing with the abundant cell codons, will become scarce (Pintó et al., 2018). Therefore, HAV has evolved avoiding the use of these codons, and thus, designing a re-coding strategy for HAV becomes extremely complicated. Instead, we adopted a classical approach of serial passaging of the virus in changing conditions of shutoff and selected the HM175-HP strain, which has adjusted the codon composition, mostly in the VP1 coding region, and in turn has acquired a more active IRES (Pérez-Rodríguez et al., 2016). Since we did only analyze the mutant swarm in a fragment of the VP1 coding region which showed an adjustment of the codon composition toward its optimization, we cannot rule out the possibility of codon frequency changes in other regions, and particularly in the region coding for the non-structural proteins. Nevertheless, mutations detected in the consensus sequence of the HP strain showed the same pattern of codon frequency changes observed in the mutant swarm of their ancestors, which did not show significant variations in the non-structural proteins coding region with respect the L0 population grown in the absence of AMD (Aragonès et al., 2010).

Production of HAV antigens remains challenging due to the low yields generated by the available cell-adapted strains. Our study shows that, the HM-175-HP strain would be a good candidate to produce a more affordable inactivated vaccine. Its IRES is more efficient than the IRES of the L0 strain in all cell substrates and is particularly active in translating genes of its same codon usage background. The use of wortmannin improves the eHAV egress and their accumulation in the supernatant, being a clear advantage for antigen production and purification. Relevant for vaccine production, is the very short half-life of wortmannin in tissue culture (Holleran et al., 2003), which would prevent toxicity issues. Although more stable than wortmannin, AMD is indeed highly sensitive to light (Budavari, 1996), providing a means for its inactivation.

Replication of the HM175-HP strain is optimal in the FRhK-4 cell line, which is not in use in vaccine manufacturing. This cell line can be adapted to grow in synthetic sera without any loss in HAV yield, consequently it could be a good cell substrate to produce HAV antigen. Similarly, it has been proposed to be used for the large-scale production of poliovirus (Dotti et al., 2017). Additionally, it has been reported to be free of tumorigenic features (Dotti et al., 2017) and of polyomavirus (Provost et al., 1984), the adventitious agent that prevented the use of the fetal rhesus cell lines for vaccine production (Klug et al., 2016). Moreover, Next-Generation-Sequencing (NGS) technologies for the detection of a broad range of viruses in combination with complete and correctly annotated viral databases may expand the range of cell substrates useful for vaccine production (Khan et al., 2020).

## Data Availability Statement

The original contributions presented in the study are included in the article/Supplementary Material, further inquiries can be directed to the corresponding author/s.

## Author Contributions

RP, SG, and AB conceived the idea and designed the study. GC-M, MC, CF, LD’A, F-JP-R, and NB performed the experimental work. RP, GC-M, and MC analyzed the data. RP wrote the manuscript. All authors approved the manuscript.

## Conflict of Interest

The authors declare that the research was conducted in the absence of any commercial or financial relationships that could be construed as a potential conflict of interest.

## Footnotes

1 www.cdc.gov/hepatitis/outbreaks/2017March-HepatitisA.htm

2 https://www.who.int/biologicals/vaccines/cell_substrates/en/

## Supplementary Material

Supplementary Figure 1 Map of the G1RCMsKpvector.
