# Supplementary figures and images for: Advances for the Hepatitis A Virus Antigen Production Using a Virus Strain With Codon Frequency Optimization Adjustments in Specific Locations
Source: Front Microbiol. 2021 Feb 18;12:642267. doi: 10.3389/fmicb.2021.642267 (PMC7935560; doi:10.3389/fmicb.2021.642267)

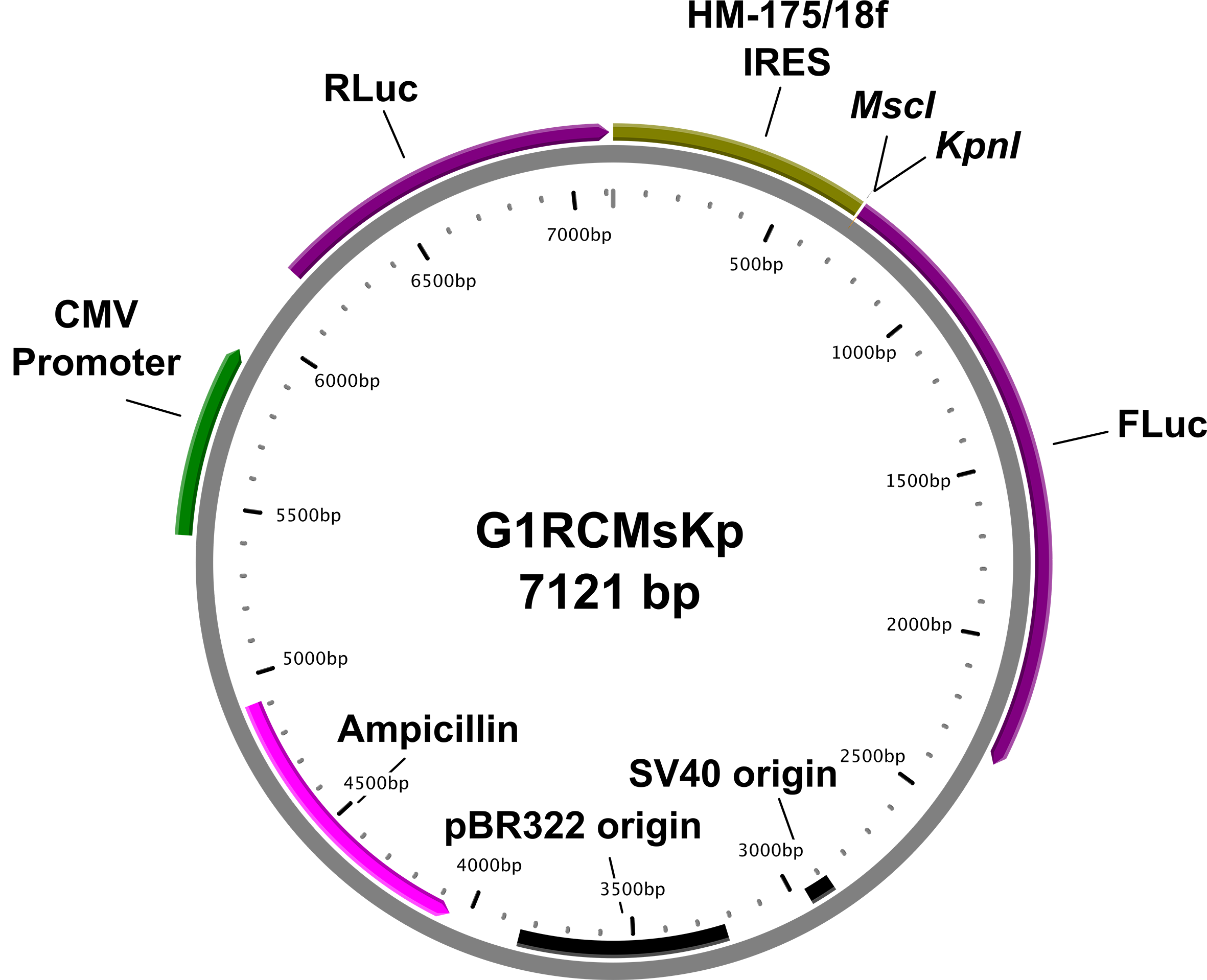

Supplement: Supplementary Figure 1 — Map of the G1RCMsKpvector. [file Image_1.TIF]
